# A Systematic Review and Meta‐Analysis of Perioperative Muscle Wasting Following Major Abdominal Surgery

**DOI:** 10.1002/jcsm.70379

**Published:** 2026-10-08

**Authors:** A. Reynolds, N. M. George, A. Papadopoulou, B. Creagh‐Brown

**Affiliations:** ^1^ Royal Surrey NHS Foundation Trust Guildford UK; ^2^ Faculty of Health and Medical Sciences University of Surrey Guildford UK

**Keywords:** major abdominal surgery, perioperative muscle wasting (POMW), sarcopenia

## Abstract

**Background:**

Muscle wasting in the perioperative period is a frequent and clinically significant complication following major abdominal surgery and is associated with adverse outcomes, including increased postoperative complications, prolonged hospitalisation and worsened mortality. Despite its prevalence, the magnitude and course of perioperative muscle wasting (POMW) remain incompletely understood, and current interventions are limited. Current definitions applied to sarcopenia do not suitably apply to cohorts undergoing major abdominal surgery, and therefore, we propose the concept of POMW, defined as POMW within 6 weeks of surgery. These systematic review and meta‐analysis aim to assess the extent of POMW and synthesise evidence on its clinical impact and potential interventions.

**Methods:**

The electronic databases MEDLINE, EMBASE and Cochrane CENTRAL were searched from their inception to June 2025. Studies with preoperative and postoperative assessments of muscle wasting and/or function within 6 weeks of major abdominal surgery were included. Methodological quality and risk of bias were assessed with the Revised Cochrane model and Newcastle–Ottawa Scale, where appropriate. Meta‐analysis was conducted using a random‐effects model, with standardised mean changes (SMCs) as effect size metrics. The primary outcome was change in skeletal muscle mass/size within 2 and 6 weeks postoperatively, and the secondary outcome was change in muscle strength.

**Results:**

A systematic review identified 45 studies (*n* = 5283 patients) evaluating perioperative changes in skeletal muscle mass/dimensions and/or function within 6 weeks of major abdominal surgery. A meta‐analysis of 17 studies (*n* = 2213 patients) was conducted, and significant reductions in muscle mass/size were observed at ≤ 2 weeks (SMC = 0.34; 95% CI: 0.19–0.49) and 3–6 weeks postoperatively (SMC = 0.39; 95% CI: 0.24–0.54). Muscle strength, primarily assessed via handgrip dynamometry, also declined significantly (SMC = 0.79; 95% CI: 0.12–1.46). Heterogeneity was high but findings remained robust in sensitivity analyses. Functional impairments and greater muscle wasting were consistently associated with poorer clinical outcomes.

**Conclusions:**

POMW is common, rapid in onset and clinically relevant. Current management strategies are inadequate. There is an urgent need for early identification, standardised monitoring and targeted interventions to prevent postoperative functional decline and improve surgical outcomes.

**PROSPERO Registration:** CRD420250606177

## 1 Introduction

Muscle wasting associated with hospitalisation is a well‐recognised phenomenon [1]. As highlighted by Shrestha et al., muscle wasting is also common during the perioperative period, yet its underlying pathophysiology remains incompletely understood and the condition is frequently under‐recognised and inadequately managed [2]. The risk is particularly high following major abdominal surgery, driven by factors including intensive care unit (ICU) admission [3], prolonged physical inactivity, inadequate nutritional intake and the systemic inflammatory response to surgery [4]. Patients who develop muscle wasting in the perioperative period experience worse postoperative outcomes [5, 6, 7], including prolonged hospitalisation [8, 9] and reduced postoperative quality of life (QoL) [9], highlighting the urgent need for improved recognition, understanding and targeted management of this complication.

Perioperative muscle wasting (POMW) is thought to be primarily driven by the catabolic response to surgical stress [10]. Comparable patterns of rapid muscle wasting have been observed in critical illness [10], suggesting shared underlying pathophysiological mechanisms [11]. In critically ill patients, skeletal muscle mass may decline by up to 2% per day during the first week of ICU admission [12], with a similarly accelerated rate of muscle wasting proposed during the early postoperative period. Despite awareness of the problem, current interventions to prevent or treat acute muscle wasting remain largely ineffective [11], highlighting the urgent need to better understand its underlying mechanisms to develop effective therapies. Enhanced Recovery After Surgery (ERAS) protocols, now implemented for most elective major abdominal surgeries, emphasise early mobilisation and nutrition optimisation to mitigate muscle wasting and reduce postoperative complications [13]. However, significant muscle wasting remains a common occurrence, and beyond early mobilisation and nutrition, no established strategies exist which specifically target the molecular and pathophysiological pathways involved.

Alongside this, there are currently no standardised approaches for its assessment or monitoring. Instead, a range of techniques are employed across clinical practice and research. The most reported methods include imaging modalities, such as computed tomography (CT) [14] and ultrasound (US) [15], which quantify changes in cross‐sectional muscle area (CSA). CT is frequently performed in patients undergoing major abdominal surgery and is thus widely available in this population, allowing for opportunistic assessment of CSA. However, its use is limited by radiation exposure and reduced accuracy in patients with a high body mass index (BMI) or significant tissue oedema [16]. In contrast, US is radiation‐free and can be performed at the bedside but remains highly operator dependent. Although both modalities provide accurate measurements of muscle size, they are generally limited to the assessment of selected muscle groups. Alternative approaches include bioelectrical impedance analysis (BIA), which estimates skeletal muscle mass by measuring the electrical resistance of body tissues to an alternating current. However, its accuracy is influenced by hydration status as well as tissue oedema and malignancy, both of which are common in patients undergoing major abdominal surgery [17, 18]. Functional measures of muscle performance are also widely used, particularly handgrip strength (HGS) [19] and knee extension strength [20]. These assessments are relatively inexpensive and suitable for bedside use; however, interpretation is complicated by substantial variation in normative values according to age, sex and ethnicity [21].

It is important to note that age and sex are key determinants of skeletal muscle and function, with ageing associated with progressive declines in muscle mass and strength [22] and sex‐related differences influencing muscle composition and metabolism [23]. Consequently, older patients are more susceptible to POMW [9], and the incidence of this is likely to rise as advances in anaesthetic and surgical care enable greater numbers of older adults to undergo major abdominal surgery [24], making it imperative to better understand this perioperative complication.

Numerous groups have proposed frameworks to better define sarcopenia and muscle wasting. In 2018, the European Working Group on Sarcopenia in Older People (EWGSOP2) updated its definition of sarcopenia to encompass reduced muscle strength, low muscle quantity or quality and impaired physical performance [22]. The group also introduced the concept of acute sarcopenia, defined as sarcopenia lasting for a period of less than 6 months [22]. Similarly, in 2019, the Asian Working Group for Sarcopenia (AWGS) defined sarcopenia as an age‐related decline in muscle mass accompanied by low muscle strength and/or impaired physical performance [25]. Importantly, the AWGS also established diagnostic cut‐off values for sarcopenia based on muscle mass, strength and physical performance, many of which have been adopted into key studies investigating POMW [25]. More recently, the Global Leadership Initiative in Sarcopenia (GLIS) met in 2024 to publish the first global conceptual definition of sarcopenia, identifying reduced muscle mass, muscle strength and muscle‐specific strength as its core components [26].

While these consensus definitions have substantially advanced the understanding and diagnosis of sarcopenia, they were primarily developed for older adults and are intended for broad application across diverse healthcare settings. Consequently, they may not fully capture the unique pathophysiological process underlying muscle wasting in the perioperative period. Patients undergoing major surgery are often younger, less frail and have fewer comorbidities than the populations for whom these definitions were developed. Furthermore, the perioperative setting provides access to detailed physiological assessments, imaging and structured follow‐up, enabling more comprehensive evaluations of muscle wasting than is typically feasible in community or general medical populations. Collectively, these differences suggest that existing definitions may not be optimally suited to characterising surgery‐induced muscle wasting.

We therefore propose the concept of POMW to describe acute skeletal muscle wasting occurring during the perioperative period following major surgery. Where the EWGSOP2 defines acute sarcopenia as developing within a 6‐month period [22], we propose that POMW should encompass muscle wasting occurring within 6 weeks of surgery. This timeframe aligns with the late postoperative phase [27], during which patients continue to experience a catabolic state, functional impairment and physiological recovery. Importantly, the 6‐week period also reflects routine surgical practice, as this is a common interval for postoperative outpatient review following major surgery, providing a practical opportunity to identify POMW. While this 6‐week threshold is necessarily pragmatic, it offers a clinically meaningful and reproducible framework for future research and clinical practice.

Recent narrative reviews, including that by Shrestha et al. [2], have highlighted the emerging importance of POMW and summarised its pathophysiology, clinical consequences and potential management strategies. However, to our knowledge, no previous systematic review and meta‐analysis have quantitatively synthesised the extent and temporal profile of POMW following major abdominal surgery.

These systematic review and meta‐analysis therefore aim to provide a comprehensive understanding and quantify the extent of POMW to define the scale of the problem and establish a foundation for future development in this field.

## 2 Methods

A systematic review was conducted according to PRISMA guidelines. Two independent reviewers (A.R. and N.G.) screened the titles and abstracts of all retrieved records, and studies not meeting predefined eligibility criteria were excluded. The full texts of potentially relevant articles were then assessed independently. Any disagreement regarding eligibility was resolved by discussion with a third reviewer. The protocol was registered with PROSPERO with registration number CRD420250606177.

### 2.1 Search Strategy

The electronic databases MEDLINE, EMBASE and Cochrane CENTRAL were searched from their inception to June 2025, using the following terms:

‘Myopenia OR Sarcopenia OR Muscle Wasting OR Muscle Loss OR Muscle Atrophy OR Muscle Depletion’.
AND
‘Oesophagectomy/Esophagectomy OR Gastrectomy OR Hepatectomy OR Pancreatectomy/Whipple's OR Pancreaticoduodenectomy OR Colectomy OR Proctosigmoidectomy OR Prostatectomy OR Cystectomy OR Laparotomy OR Hartmann's OR Radical OR Nephrectomy OR Abdominal Resection OR Abdominal Surgery OR Open Aortic Abdominal Aneurysm OR Surg*/Operat* OR Radical Hysterectomy OR Pelvic Exenteration OR Debulking surgery OR Cytoreductive surgery’.

### 2.2 Inclusion and Exclusion Criteria

Studies were included if they quantified perioperative changes in skeletal muscle mass/dimensions and/or strength/function in adults undergoing major abdominal surgery, with both preoperative and postoperative measurements reported within 6 weeks of surgery. No minimum threshold of postoperative decline or requirement for statistical significance within individual studies was applied as an eligibility criterion, enabling inclusion of all studies reporting quantitative perioperative muscle changes. Randomised controlled trials, cohort studies, case–control studies, case reports, poster presentations and conference abstracts were eligible. Studies were excluded if they did not report quantitative outcomes of skeletal muscle mass/dimensions or strength/function, if outcomes could not be extracted or if they were nonhuman, nonclinical, paediatric or bariatric studies. For studies reporting measurements at multiple postoperative time points, including both within and beyond the 6‐week postoperative window, only data collected within the first 6 weeks after surgery were extracted and included in the primary analysis. For studies that evaluated interventions intended to influence POMW, only data from the standard care control groups were extracted for the analysis to ensure comparability.

Two reviewers (N.G. and A.R.) independently extracted data from each study using a predefined template. Extracted data included study design, sample size, patient characteristics, type of surgery, method of muscle assessment and timing of measurements. For studies reporting results separately by subgroups (e.g. sex), the weighted mean and pooled standard deviation (SD) were calculated and included in the analysis. Where data were reported as median and interquartile range or range, mean and SD were estimated using the quantile estimation method. If results were presented only in graphical form, numerical values were extracted using WebPlotDigitiser.

The primary outcome was change in skeletal muscle mass/size within 2 and 6 weeks postoperatively, measured by imaging (CT, US, dual‐energy X‐ray absorptiometry [DXA]) or BIA. Postoperative assessments were grouped into ≤ 2 and 3–6 weeks to distinguish the acute postoperative period from the later recovery phase. These intervals also reflected the clustering of postoperative assessment time points across the included studies, allowing clinically meaningful pooling while maximising the number of studies contributing to each analysis. The secondary outcome was change in muscle strength, mostly assessed using handgrip dynamometry.

A random‐effects model was applied given the anticipated variability in muscle mass and strength measurement methods. The effect size was expressed as the mass/dimensions standardised mean change (SMC), calculated using raw score standardisation with pooled SD and incorporating Hedges small‐sample correction. Positive SMC values indicate postoperative decline relative to baseline. Sampling variances were estimated using the heteroscedastic standardised mean change estimator (SMCRPH) that allows unequal population variances at baseline and follow‐up and incorporates within‐subject correlations. Where correlation coefficients between preoperative and postoperative measures were available, these were incorporated. For studies without reported correlations, values were imputed, and sensitivity analyses were performed across a plausible range (*r* = 0.3–0.9) to assess robustness of findings. Heterogeneity was quantified using the *I* ^2^ statistic and tau^2^. Prediction intervals were calculated to estimate the range of effects in future comparable studies. Effect sizes were interpreted according to Cohen's convention: 0.2 as small, 0.5 as moderate and 0.8 as large. Statistical significance was defined as *p* < 0.05. All statistical analyses were conducted using R (version 4.5.1).

### 2.3 Assessment of Methodological Quality

Methodological quality and risk of bias were assessed using the Newcastle–Ottawa Scale for observational studies (Table S1) and the Revised Cochrane risk‐of‐bias tool (RoB 2) for randomised trials (Table S2).

## 3 Results

### 3.1 Study Selection

The database search identified 3695 records which were reduced to 2133 following duplication removal. After screening, 45 studies comprising 5283 patients met the inclusion criteria for the systematic review (Table S3). The study selection process is detailed in the PRISMA flow diagram (Figure 1). Following this, 17 studies (*n* = 2213) were suitable for inclusion into the quantitative meta‐analysis (Figures 2, 3, 4).

**FIGURE 1:**
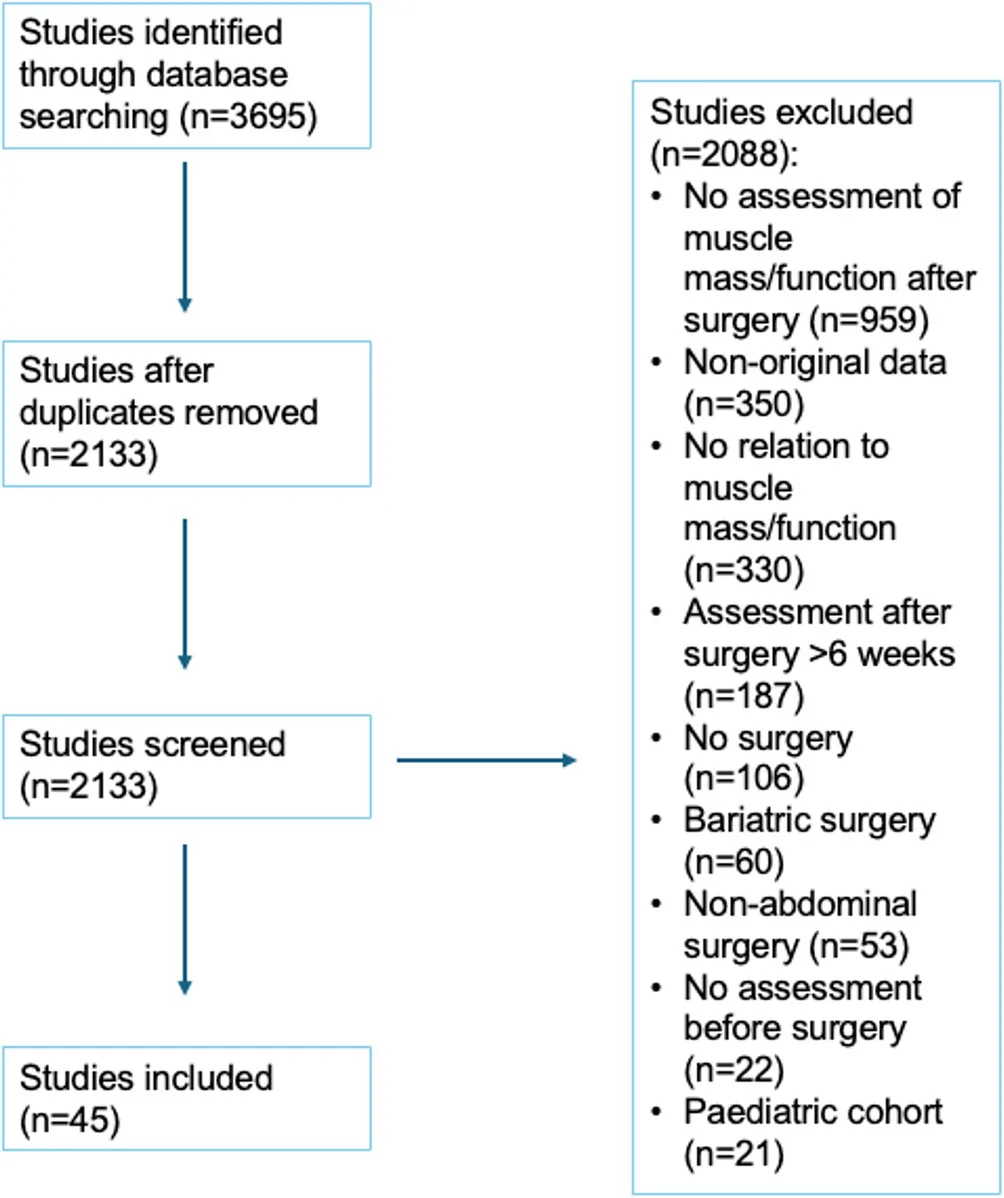
PRISMA flow diagram.

**FIGURE 2:**
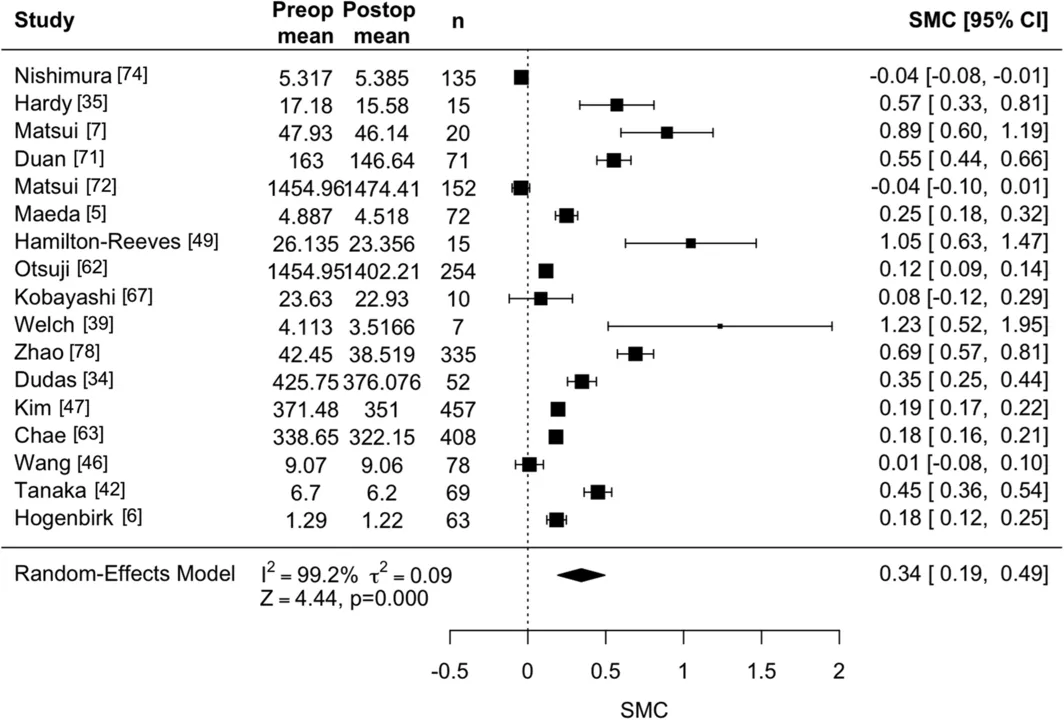
Forest plot of meta‐analysis of muscle wasting within 2 weeks postoperatively.

**FIGURE 3:**
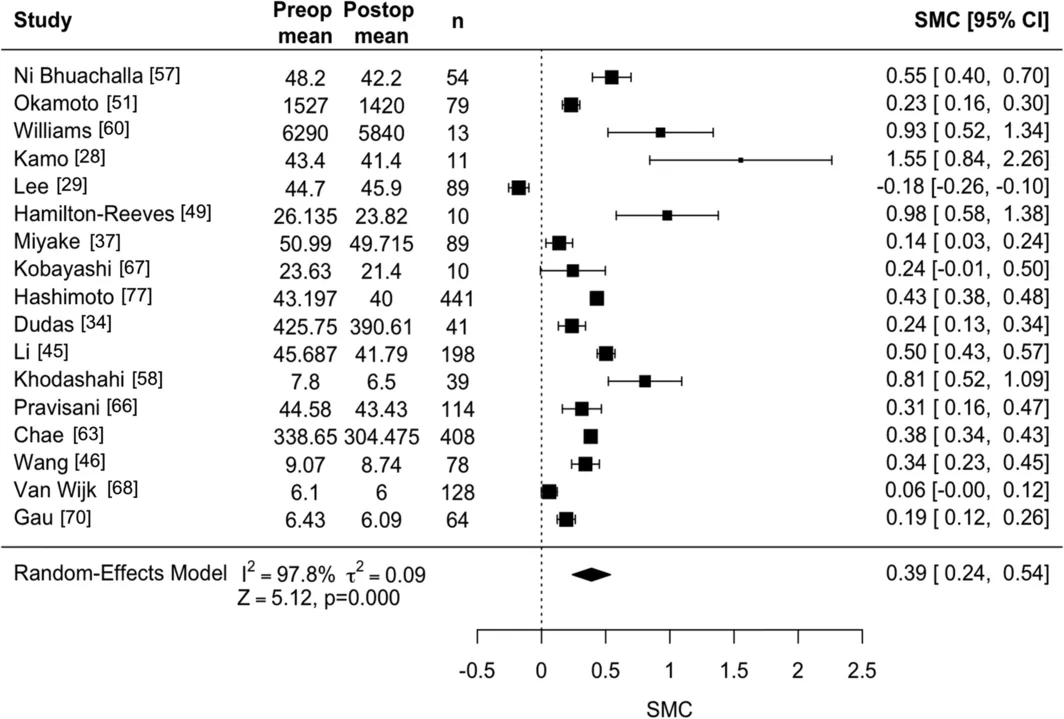
Forest plot of meta‐analysis of muscle wasting within 1 month postoperatively.

**FIGURE 4:**
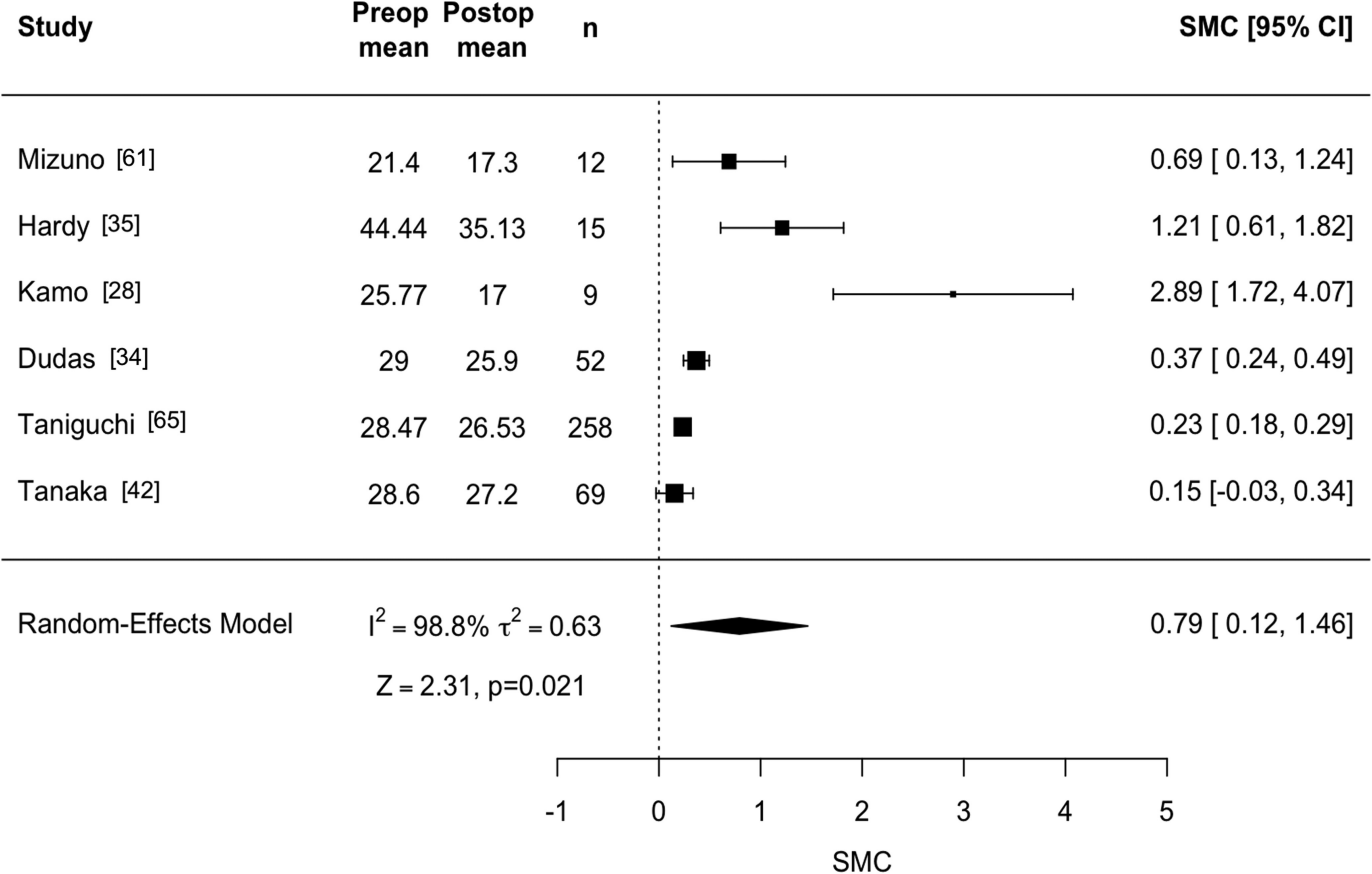
Forest plot of meta‐analysis of grip strength change postoperatively.

### 3.2 Study Characteristics

Included studies were published between 1999 and 2025 and encompassed a broad range of major abdominal surgeries, including hepatopancreatobiliary (HPB), upper gastrointestinal (GI), colorectal and urological procedures. Study designs included prospective observational cohorts (*n* = 21), retrospective analyses (*n* = 16) and randomised controlled trials (*n* = 8). The reported mean age of participants ranged from 54 to 72 years.

Methods used to assess skeletal muscle mass/size varied considerably: CT was the most frequent (*n* = 23), followed by BIA (*n* = 11), US (*n* = 4) and DXA (*n* = 2). Muscle strength was predominantly assessed by HGS (*n* = 9). The majority of preoperative assessments were performed within 1 month before surgery (Table S3). Postoperative assessment intervals ranged from the immediate postoperative period to 6 weeks and were grouped for meta‐analysis as ≤ 2 (early postoperative) and 3–6 weeks (intermediate‐late postoperative).

#### 3.2.1 Pooled Analysis of Skeletal Muscle Mass/Size

##### 3.2.1.1 About *≤* 2Weeks Postoperatively

Seventeen studies involving 2213 patients reported quantitative changes in skeletal muscle mass/dimensions within 2 weeks of surgery. The pooled SMC demonstrated a significant reduction in muscle mass/dimensions (SMC = 0.34; 95% CI 0.19–0.49; *p* < 0.001) (Figure 2). The significance of these findings was confirmed using a range of imputed correlation values (*r* = 0.3–0.9), *p* < 0.0001. The 95% prediction interval was −0.27 to 0.95. Heterogeneity was high (*I* ^2^ = 99%), and leave‐one‐out sensitivity analysis did not reveal any outliers. To explore sources of heterogeneity, a subgroup analysis restricted to nine studies that used CT for skeletal muscle measurement was performed. The direction and magnitude of effect were consistent with the primary analysis (SMC = 0.28; 95% CI 0.09–0.48; *p* < 0.0001) across a range of imputed correlation values (*r* = 0.7–0.9). However, statistical heterogeneity remained substantial (*I* ^2^ = 98%). Further subgroup analyses were performed according to anatomical area assessed by CT imaging. Both psoas muscle area (six studies) and L3 skeletal muscle area (three studies) demonstrated significant reduction postoperatively; SMC = 0.11, 95% CI 0.01–0.21, *p* = 0.03 and SMC 0.66, 95% CI 0.52–0.81, *p* < 0.0001, respectively. Statistical heterogeneity was moderate to considerable (*I* ^2^ = 94.7% and 52%, respectively). Similarly, US‐based assessment of thigh skeletal muscles demonstrated a statistically significant reduction postoperatively (four studies, SMC 0.44, 95% CI 0.16–0.72, *p* = 0.002, *I* ^2^ = 90%).

### 3.3 About 3–6 Weeks Postoperatively

At approximately 1 month after surgery, 17 studies comprising 1882 participants reported perioperative muscle mass/dimension changes. The pooled SMC again demonstrated a significant decline (SMC = 0.39; 95% CI 0.24–0.54; *p* < 0.001) (Figure 3). The significance of the findings was robust across a range of imputed correlation values (*r* = 0.3 to 0.9), *p* < 0.0001. The 95% prediction interval ranged from −0.21 to 0.99. Heterogeneity remained high (*I* ^2^ = 97.8%), and leave‐one‐out sensitivity analysis did not reveal any outliers. To explore sources of heterogeneity, a similar subgroup analysis was performed restricted to 10 studies that used CT for skeletal muscle measurement. The direction and magnitude of effect were consistent with the primary analysis (SMC = 0.28; 95% CI 0.12–0.45; *p* < 0.0001) across a range of imputed correlation values (*r* = 0.7 to 0.9). However, statistical heterogeneity remained substantial (*I* ^2^ = 96%). Influence diagnostics identified two studies (Kamo et al. [45] and Lee et al. [46]) contributing disproportionately to the between‐study heterogeneity. However, leave‐one‐out analysis demonstrated that omission of any individual study did not significantly alter the pooled effect estimate, indicating that the findings were robust. Further subgroup analysis according to the measured anatomical area demonstrated significant reduction in both psoas muscle area (four studies, SMC 0.22, 95% CI 0.09–0.35, *p* = 0.001) and skeletal muscle area at L3 level (6 studies, SMC 0.38, 95% CI 0.02–0.74, *p* = 0.038). Statistical heterogeneity remained substantial, *I* ^2^ = 93.6% and 98.6%, respectively. A comparable reduction was observed in BIA‐based assessment (four studies, SMC 0.48, 95% CI 0.26–0.69, *p* < 0.001, *I* ^2^ = 78.8%). Similarly, exploratory subgroup analysis of patients undergoing liver transplant surgery demonstrated a significant reduction in skeletal muscle postoperatively (four studies, SMC 0.68, 95% CI 0.21–1.14, *p* = 0.004, *I* ^2^ = 95.9%).

### 3.4 Pooled Analysis of Muscle Strength

Six studies including 417 patients assessed perioperative changes in muscle strength, predominantly using HGS. Pooled analysis demonstrated a significant reduction in muscle strength following surgery (SMC = 0.79; 95% CI 0.12–1.46; *p* = 0.02) (Figure 3). However, heterogeneity was substantial, and the 95% prediction interval was wide (−0.90 to 2.49), indicating considerable between‐study variability in the magnitude of effect. While the overall direction of effect supports the presence of clinically relevant perioperative muscle weakness, these findings should be interpreted with caution given the limited number of studies and imprecision of estimates.

## 4 Discussion

### 4.1 Qualitative Discussion

These systematic review and meta‐analysis demonstrate that POMW is a common and clinically meaningful phenomenon following major abdominal surgery. Across 45 studies involving 5283 patients, reductions in muscle mass, size and function were consistently observed within the first 6 weeks postoperatively, regardless of the modality used to assess them. Techniques including CT imaging, US, DXA scans, BIA and HGS all demonstrated significant perioperative declines, suggesting that POMW is a reproducible finding across different measurement methods. Importantly, several studies reported associations between POMW and adverse clinical outcomes, including higher rates of postoperative complications and reduced survival. Together, these findings highlight that POMW is not only prevalent but also clinically important, reinforcing the need for strategies aimed at early identification, targeted intervention and prevention of POMW.

#### 4.1.1 Surgical Procedures

A broad spectrum of major abdominal surgical procedures was represented among the included studies. HPB surgery was the most common, accounting for 19 studies. This was followed by upper GI surgery in 15 studies, colorectal surgery in five studies and urological surgery in two studies. The remaining studies involved pooled populations undergoing a mix of major abdominal operations. Muscle wasting was consistently observed across all surgical groups, irrespective of specialty or specific procedure. This uniformity underscores that POMW is a widespread and generalisable phenomenon following major abdominal surgery, rather than being confined to a single surgical domain. Consequently, this highlights the importance of early recognition and intervention strategies across the perioperative pathway, irrespective of surgical type.

All included studies were conducted in elective surgical populations. Given the preoperative preparation and optimisation that many patients undergo before elective major surgery [56], baseline muscle function may have been enhanced, potentially attenuating the extent of POMW. In contrast, patients undergoing emergency major abdominal surgery do not benefit from this period of optimisation and frequently present with marked inflammation and physiological derangement, factors that may predispose them to greater POMW. Future studies should therefore investigate the incidence and severity of POMW following emergency surgery and compare these findings with elective cohorts to determine whether this population is at increased risk, thereby facilitating earlier identification and targeted intervention.

#### 4.1.2 Assessment Modalities

There was considerable heterogeneity in how perioperative muscle mass/dimensions were assessed across the included studies. CT imaging was the most frequently employed modality, used in 23 studies. In most cases, CT was utilised to quantify specific muscle groups, most commonly at the L3 vertebral level to provide standardisation. Muscle measurements were typically compared preoperatively and postoperatively to determine the degree of muscle wasting. The widespread adoption of CT for these purposes likely reflects its practical advantages in this patient population with routine perioperative imaging providing readily available data and thus minimising additional procedures, patient inconvenience and radiation exposure. However, CT‐based muscle assessment also has notable limitations, including reduced image quality and accuracy in patients with a high BMI alongside a chance of postoperative oedema distorting measurements, which may have impacted the accuracy of results [16]. BIA was the second most common modality, used in 11 studies. BIA estimates body composition by analysing tissues resistance to an electrical current, providing a rapid, noninvasive and relatively inexpensive method of body composition assessment. However, BIA is also prone to systematic error and prediction bias [16]. Results have been observed to be influenced by factors such as hydration status [17, 18], tissue oedema and malignancy, all factors which would be important to consider in this patient population, potentially compromising the accuracy of results [18]. Importantly, none of the included studies employed both BIA and CT within the same cohort, precluding a direct comparison between these modalities. US imaging was used in four studies, all of which assessed CSA of the anterior thigh musculature. US offers a radiation‐free, bedside method for evaluating muscle size, and reliability of rectus femoris measurements via US assessment is well‐established [57]. The increasing availability of portable US machines makes this an appealing option for perioperative muscle assessment. As well as muscle size quantification, US can also evaluate muscle quality through echogenicity [58], a method shown to be feasible in critically ill patients [59]. However, US is operator dependent, which introduces potential measurement bias; this can be mitigated by standardised protocols or single‐operator assessments, as demonstrated by Dudas et al. [37]. Overall, the predominance of CT imaging suggests that it will likely remain the principal modality for assessing POMW in this population. Nonetheless, the increasing adoption of US, driven by its bedside applicability and safety, offers a promising complementary tool. In contrast, while BIA offers practical advantages, its susceptibility to physiological fluctuations in the perioperative period may limit its utility in accurately capturing acute changes in muscle mass.

#### 4.1.3 Timings

A substantial proportion of studies demonstrated that POMW develops rapidly after major abdominal surgery. A total of 23 studies assessed POMW within 10 days postoperatively, of which 21 reported measurable declines in muscle mass/dimensions during this early period. Notably, some studies identified reductions within just 5 days of surgery. For example, Maeda et al. reported a 4.4% decrease in total psoas muscle index (TPI) 3 days following oesophagectomy [5], while Hardy et al. observed a 9.16% reduction in the CSA of the vastus lateralis within 5 days of open colorectal resection [29]. These findings highlight that clinically relevant muscle wasting occurs almost immediately after surgery, reinforcing the need for early, targeted interventions if they are to be maximally effective in mitigating muscle wasting.

Importantly, muscle wasting was also frequently observed at 1 month postoperatively, illustrating the sustained impact of the perioperative catabolic state. Longer term data underscore the prolonged recovery trajectory; Kaido et al. reported that skeletal muscle mass did not return to baseline until 12 months after living‐donor liver transplantation [60], while Miyake et al. demonstrated persistent deficits in skeletal muscle area at the level of L3 up to 24 months postoperatively [47]. Taken together, these findings indicate that POMW develops rapidly in the immediate postoperative period and may persist for months to years thereafter. This highlights the critical need for early identification and timely intervention, potentially requiring preventative rather than reactive measures if meaningful mitigation of POMW is to be achieved.

#### 4.1.4 Effects on Muscle Function

Perioperative changes in muscle function were frequently assessed across the included studies, most commonly through measures of contractile muscle strength. This is a crucial dimension of POMW, as many of the adverse clinical outcomes observed are likely driven by functional decline. HGS was the most frequently used functional assessment, being employed in nine studies. Although HGS is a practical and widely used bedside measure, it is susceptible to considerable variability arising from factors such as the device used, assessment of the dominant versus nondominant hand, the number of attempts performed, patient positioning and hand size [61]. Consequently, methodological variation between studies may have influenced the reported findings. Across all but one study, significant perioperative declines in HGS were observed. The exception was Welch et al., who reported no significant change in HGS in patients over 65 years undergoing elective colorectal surgery [35]. However, this was a small pilot study with only seven participants, meaning the lack of statistical power likely contributed to the absence of a detectable effect. The consistency of findings across the remaining studies supports a robust association between surgery and reduced muscle strength in the early postoperative period.

In addition to strength testing, physical activity levels were used as indicators of muscle function. Dudas et al. employed actigraphy to monitor early postoperative mobility, demonstrating that patients with < 10% loss of rectus femoris cross‐sectional area (RFCSA) had significantly higher activity counts in the first two postoperative days compared to those with > 10% loss [37]. A positive correlation between activity levels and RFCSA preservation was also observed. These findings reinforce the central role of early mobilisation in mitigating POMW, in line with the principles of the ERAS protocols [62, 63].

Five studies assessed both muscle mass/size and function concurrently [35, 37, 41, 45, 60]. All demonstrated parallel declines in both parameters, apart from the small feasibility study carried out by Healy et al. [64]. This alignment between mass/dimensions and function suggests that POMW is a reliable surrogate for functional decline. Interestingly, Kaido et al. found that HGS returned to baseline within 6 months postoperatively, whereas muscle mass recovery lagged to 12 months [60], indicating that functional recovery may precede structural recovery.

Consistent perioperative declines in muscle function across studies underscore its clinical importance. Ultimately, it is the loss of function, rather than mass alone that directly impacts patient QoL, independence and recovery trajectory. Therefore, further high‐quality studies are needed to more precisely investigate this loss of function in the perioperative period and to explore targeted strategies for functional preservation and rehabilitation.

#### 4.1.5 Clinical Consequences

As demonstrated across multiple studies, POMW is consistently associated with a worse clinical prognosis. Several studies included in this review identified clear links between the extent of POMW and adverse short‐ and long‐term outcomes.

Huang et al. reported that patients experiencing significant POMW, > 10% relative to preoperative baseline, following gastrectomy for gastric cancer had a markedly higher rate of postoperative complications [9]. Similarly, Shimoda et al. found that after oesophagectomy, greater degrees of muscle wasting were associated with increased postoperative morbidity, particularly anastomotic leakage and reduced long‐term survival compared to patients with less pronounced POMW [65]. Li et al. further observed that skeletal muscle wasting following gastrectomy was associated with poorer overall survival [49], while Lee et al. demonstrated that the restoration of preoperative muscle size within 24 weeks of pancreatic resection was an independent prognostic factor for survival [46].

Beyond morbidity and mortality outcomes, POMW also has a significant effect on patient‐centred measures such as QoL. Huang et al. identified that greater degrees of muscle wasting were associated with poorer postoperative QoL [9], while Wang et al. demonstrated that excessive skeletal muscle wasting following oesophagectomy significantly impaired QoL recovery [40]. These findings further underline that the consequences of POMW extend beyond clinical endpoints and can affect patient experience and overall well‐being.

Taken together, these findings highlight that POMW is not merely a transient physiological response but rather a clinically significant marker of adverse outcomes. The persistence of its effects on morbidity, mortality and QoL emphasises the sustained impact of the postoperative catabolic state. Early recognition and targeted management of muscle wasting therefore represent key opportunities to enhance recovery, reduce complications and improve long‐term survivorship and QoL.

#### 4.1.6 POMW in Healthy Cohorts

Kim et al. demonstrated, in a retrospective analysis of 457 healthy adult living donors who underwent right lobe hepatectomy, that significant POMW occurred within the first postoperative week, with recovery by 3 months [38]. This is an important finding because this cohort provides a unique opportunity to evaluate the effects of major surgery in an otherwise healthy population, thereby reducing the influence of underlying disease and highlighting the significant impact surgery itself can have. However, it is important to consider that hepatic resection and the metabolic demands of liver regeneration are likely to have contributed to the observed muscle wasting, although the extent of their contribution remains uncertain.

Nonetheless, living liver donors represent a valuable model for investigating POMW following major surgery in patients without significant comorbidity. To our knowledge, the study by Kim et al. [38] remains the only assessment in this population, highlighting an important gap in knowledge within the perioperative field. Further research in such cohorts could not only improve care for living organ donors but also enhance our understanding of the mechanisms underlying POMW and better isolate the effects of surgery itself.

#### 4.1.7 Interventional Evidence

This review identified 12 studies that assessed the impact of specific interventions on POMW. Nutritional interventions were the most frequently studied, featuring in seven of these trials. The approaches investigated included carbohydrate loading [66], immunonutrition [32], amino acid supplementation [43, 67] and β‐hydroxy‐β‐methylbutyrate (HMB)‐enriched formulas [45]. Although results were mixed, most studies demonstrated that such interventions resulted in a modest reduction in muscle wasting. For example, Hamilton‐Reeves et al. found that perioperative immunonutrition reduced skeletal muscle wasting at 2 weeks from 16.9% in the control group to 7.04% in the intervention group [32]. Similarly, Kamo et al. reported that HMB‐enriched supplementation improved both skeletal muscle preservation and HGS at 1 month following liver transplantation compared to the control group [45]. However, in the study by Healy et al., postoperative administration of eicosapentaenoic acid (EPA)‐enriched enteral feed did not result in a significant difference in muscle mass or HGS compared with the control group at 1 month after oesophagectomy [64].

Two studies evaluated neuromuscular electrical stimulation (NMES) as a non‐nutritional strategy to attenuate POMW [29, 68]. In a split‐body randomised controlled trial following open colorectal resection, Hardy et al. demonstrated that NMES significantly reduced quadriceps muscle wasting compared with the untreated limb [29]. Similarly, Tsutsui et al. reported that NMES applied to the quadriceps following living‐donor liver transplantation prevented postoperative reduction in both quadriceps and psoas muscle areas at 1 month, relative to the control group, implying potential systemic benefits of NMES [68].

In addition, Nose et al. explored the use of postoperative ghrelin administration following oesophagectomy, finding that patients who received ghrelin exhibited significantly less loss of erector spinae muscle 1 week after surgery compared with those given placebo [69].

Collectively, these early studies highlight the early potential for targeted perioperative interventions to mitigate POMW. However, the existing evidence base is limited by small sample sizes, heterogeneous methodologies and short follow‐up periods. Larger, high‐quality randomised controlled trials are therefore required to further clarify the efficacy of these interventions and explore their potential use in the perioperative period.

### 4.2 Quantitative Discussion

The presented meta‐analysis synthesised evidence from 17 studies evaluating changes in skeletal muscle mass/size and function following major surgery. The results demonstrate a statistically significant reduction in skeletal muscle mass/size by approximately 0.34 and 0.39 SDs within 2 weeks and 1 month, respectively, postoperatively. Although modest in size, this effect size corresponds to a 13‐ and 15‐percentile‐point downward shift of skeletal muscle mass/size within 2 weeks and 1 month, respectively, postoperatively. Muscle strength, primarily assessed using handgrip dynamometry, also declined significantly in the perioperative period. These findings indicate that quantitative and functional measures of skeletal muscle, although distinct entities, are both adversely affected in the early postoperative course.

Most prior meta‐analyses in major abdominal surgery have focused on baseline sarcopenia as a prognostic factor for postoperative complications and mortality [70, 71, 72]. By contrast, the present findings support the concept that muscle wasting occurs dynamically in the immediate postoperative period, even among patients without pre‐existing sarcopenia. This highlights the need for perioperative interventions aimed at preserving skeletal muscle, regardless of baseline status.

The magnitude of decline in muscle mass/size and strength observed in this meta‐analysis is comparable to that described in critical illness [12] and prolonged immobilisation, albeit over a shorter perioperative timeframe. This suggests that surgical insult may precipitate an acute catabolic state that accelerates skeletal muscle deterioration, as demonstrated in critically unwell patients [10].

Although L3 skeletal muscle measurements are generally considered a more representative CT‐based measure of whole‐body muscle mass, our subgroup analyses did not demonstrate a consistent reduction in heterogeneity when studies were stratified according to anatomical site. Both psoas and skeletal muscle measurements identified statistically significant postoperative muscle loss indicating that the observed decline is robust across CT measurement techniques. The persistence of heterogeneity within anatomical subgroups suggests that variability is multifactorial and likely influenced by surgical procedure, patient population and perioperative care rather than determined by anatomical site alone.

It is important to note that measurement precision and reproducibility may vary, not only between different imaging modalities but also within the same technique according to acquisition protocols, analysis software and reporting methodology. Consequently, small reported changes may approach the limits of precision in some studies, representing a potential source of between‐study variability.

The observed effect sizes of the perioperative changes in muscle mass/size and function were small to moderate by conventional benchmarks and remained consistent across both short‐term and 1‐month follow‐up intervals. Statistical heterogeneity was substantial, likely reflecting differences in surgical procedures and perioperative care protocols, and was further influenced by the narrow confidence intervals of several large studies that amplified the relative between‐study variance. The 95% prediction intervals were wide and crossed zero, suggesting that the magnitude of effect in future individual studies may vary substantially. However, sensitivity analysis demonstrated stability of the pooled estimates across a wide range of imputed correlation coefficients. Taken together, despite the substantial variability, our findings provide a useful quantitative summary of perioperative skeletal muscle changes and complement the qualitative systematic review. While the results should be interpreted with caution, the analysis offers valuable insights for future studies.

#### 4.2.1 Strengths and Limitations

Several methodological strengths can be noted. Given the variability in muscle mass and dimensions assessment methods, we chose a random effects model, applied Hedges' small‐sample correction to reduce bias, accounted for heteroscedasticity across the different time points and confirmed our findings using sensitivity and subgroup analyses across a wide range of plausible correlation coefficients.

However, limitations must be acknowledged. There was considerable variation in muscle mass, size and strength measurement methods, and there was significant heterogeneity across studies. This likely reflects clinical and methodological differences between the studies, including variation in patient cohorts, procedures, timing of postoperative assessments and methods used to quantify muscle wasting. The heterogeneity remained high in analyses restricted to CT‐based measures, suggesting that differences in CT‐derived muscle outcomes (e.g. SMI, CSA or psoas measurements), imaging protocols and assessment time points may also contribute. These findings highlight the need for greater standardisation of outcome definitions and measurement methodologies in future studies of POMW. In addition, most included studies did not report modality‐specific measurement precision or minimal detectable change, preventing assessment of whether small postoperative changes exceeded the limits of measurement error. This should be considered when interpreting studies reporting relatively small changes in muscle mass or dimensions.

Reporting bias is another potential limitation of this review. Studies demonstrating significant POMW may have been more likely to be published or to report detailed muscle‐related outcomes than those showing little or no postoperative change. Furthermore, selective outcome reporting within individual studies cannot be excluded, and the findings should therefore be interpreted with caution. Selection bias is also an important consideration, particularly in retrospective studies in which postoperative imaging was performed for clinical rather than research purposes. Patients undergoing additional postoperative imaging may have experienced complications or greater illness severity, making them more susceptible to muscle wasting and potentially leading to an overestimation of the true magnitude of POMW.

Furthermore, the timing of preoperative muscle assessment relative to surgery was inconsistently reported across the included studies (Table S1). Although most studies were assessed within 1 month before surgery, several did not specify the timing. Consequently, it was not always possible to distinguish postoperative muscle changes attributable to surgical insult from those related to underlying disease progression or preoperative physiological change.

Additionally, given the complexity of the surgical procedures included, it is likely that many patients required admission to an ICU or surgical high‐dependency unit (HDU) during the early postoperative period for monitoring and higher level care, meaning the contribution of ICU‐AW should be considered. Consequently, a proportion of the observed muscle wasting may have been attributable to ICU‐AW, making it difficult to distinguish POMW related to surgery alone from that associated with ICU admission.

A small number of conference presentations and abstracts were included in the analysis that had not undergone full peer review [42, 50] which may have affected quality assessment and introduced additional risk of bias.

### 4.3 Clinical Implications

This review highlights that the proposed concept of POMW, defined as muscle wasting within 6 weeks of surgery, is both common and clinically significant, with measurable declines in skeletal muscle mass, size and function occurring during this period. These changes are statistically significant and comparable to muscle wasting observed in critical illness [12], emphasising the severity of the catabolic response to surgical stress.

Importantly, the observed reductions in muscle mass/dimensions and strength have been consistently associated with adverse postoperative outcomes, including increased complication rates, prolonged hospitalisation, delayed recovery and reduced long‐term survival. These data reinforce the need to shift perioperative care from a solely surgical focus to a more integrated approach that includes early identification, monitoring and mitigation of muscle wasting.

Routine incorporation of objective muscle assessments, such as CT‐based skeletal muscle index or grip strength testing, into perioperative protocols and diagnostic criteria may allow for earlier detection of high‐risk patients. Furthermore, the findings support the use of targeted multimodal interventions, including prehabilitation, early mobilisation and nutritional supplementation, even in patients without pre‐existing sarcopenia.

Given the heterogeneity of surgical populations and recovery trajectories, further research should aim to define personalised thresholds for clinically meaningful muscle wasting and to evaluate the effectiveness of muscle‐preserving interventions in randomised trials. This analysis of the current evidence suggests that perioperative muscle health is a modifiable and under‐recognised determinant of surgical outcome and should become a routine target of perioperative optimisation strategies.

## 5 Conclusion

We propose the concept of POMW as an important and prevalent occurrence following major abdominal surgery. This systematic review demonstrates that POMW and impaired muscle function are near‐universal consequences of major abdominal surgery. Loss of muscle mass, size and function is consistently associated with increased short‐ and long‐term morbidity, mortality and a reduced QoL, highlighting its significant clinical relevance.

Although interventional research in this area remains at a very early stage, emerging evidence suggests that some interventions may help mitigate POMW. Future work in this field should focus on improving awareness and understanding of the rapid onset and severity of POMW and on developing, validating and implementing targeted interventions to prevent its occurrence and improve postoperative outcomes.

## Conflicts of Interest

The authors declare no conflicts of interest.

## Supporting information


**Table S1:** Risk of bias assessment for observational studies using the Newcastle–Ottawa Scale.
**Table S2:** Risk of bias assessment for randomised controlled studies using the Cochrane–RoB scale.
**Table S3:** Characteristics of included studies, listed in chronological order of publication.

## Data Availability

Data sharing is not applicable to this article as no datasets were generated or analysed during the current study.
