# Supplementary material for: A Systematic Review and Meta‐Analysis of Perioperative Muscle Wasting Following Major Abdominal Surgery
Source: J Cachexia Sarcopenia Muscle. 2026 Oct 8;17(5):e70379. doi: 10.1002/jcsm.70379 (PMC13649356; doi:10.1002/jcsm.70379)
Supplement: Supplementary file 1 [file JCSM-17-e70379-s001.docx]

**Supplemental Tables**

| **Risk of Bias – Observational Studies** | | | | |
| --- | --- | --- | --- | --- |
|  | **Selection** | **Comparability** | **Outcome** | **Overall** |
| Williams et al., 2012 [60] | ++++ | ++ | +++ | Low |
| Ni Bhuachalla et al., 2015 [57] | +++ | + | ++ | Low |
| Kaido et al., 2016 [36] | ++++ | ++ | +++ | Low |
| Mizuno et al., 2016 [61] | ++++ | + | ++ | Moderate |
| Miyake et al., 2017 [37] | ++++ | + | +++ | Low |
| Otsuji et al., 2017 [62] | ++++ | ++ | +++ | Low |
| Huang et al., 2017 [9] | ++++ | + | +++ | Low |
| Chae et al., 2018 [63] | ++++ | - | +++ | Moderate |
| Chae at al., 2018 [64] | ++++ | ++ | +++ | Low |
| Taniguchi et al., 2019 [65] | ++++ | + | ++ | Moderate |
| Welch et al., 2019 [39] | ++++ | + | ++ | Moderate |
| Kim et al., 2019 [47] | ++++ | + | +++ | Low |
| Pravisani et al., 2019 [66] | ++++ | ++ | +++ | Low |
| Kobayashi et al., 2019 [67] | +++ | + | +++ | Low |
| Shimoda et al., 2020 [44] | ++++ | + | +++ | Low |
| Maeda et al., 2020 [5] | ++++ | + | +++ | Low |
| Lee et al., 2020 [29] | ++++ | + | +++ | Low |
| van Wijk et al., 2021 [68] | ++++ | + | +++ | Low |
| Cha et al., 2021 [69] | +++ | - | ++ | High |
| Gau et al., 2022 [70] | ++++ | ++ | +++ | Low |
| Duan et al., 2021 [71] | ++++ | + | +++ | Low |
| Matsui et al., 2021 [7] | ++++ | + | +++ | Low |
| Tanaka et al., 2022 [42] | ++++ | + | ++ | Moderate |
| Matsui et al., 2022 [72] | ++++ | + | ++ | Moderate |
| Kikuchi et al., 2022 [73] | ++++ | + | ++ | Moderate |
| Wang et al., 2022 [46] | +++ | + | ++ | Moderate |
| Hogenbirk et al., 2023 [6] | +++ | + | ++ | Moderate |
| Nishimura et al., 2023 [74] | ++++ | + | ++ | Moderate |
| Osaki et al., 2023 [75] | ++++ | + | ++ | Moderate |
| Okamoto et al., 2024 [51] | ++++ | + | +++ | Low |
| Khodashahi et al., 2024 [58] | +++ | + | ++ | Moderate |
| Koh et al., 2024 [76] | +++ | - | +++ | Moderate |
| Hashimoto et al., 2025 [77] | ++++ | ++ | +++ | Low |
| Kikuchi et al., 2025 [50] | ++++ | - | +++ | Low |
| Dudas et al., 2025 [34] | +++ | + | ++ | Moderate |
| Li et al., 2025 [45] | ++++ | ++ | +++ | Low |
| Zhao et al., 2025 [78] | ++++ | ++ | +++ | Low |

Supplemental Table 1 - Risk of bias assessment for observational studies using the Newcastle-Ottawa Scale.

| **Risk of Bias- Randomised Trials** | | | | | | | |
| --- | --- | --- | --- | --- | --- | --- | --- |
|  |  | **D1** | **D2** | **D3** | **D4** | **D5** | **Overall** |
| Kissmeyer-Nielsen at al., 1999 [59] | RoB - RCT | Low | Low | Some | Low | Some | Some |
| Henriksen et al., 2003 [48] | RoB - RCT | Low | Low | Some | Low | Some | Some |
| Healy et al., 2017 [43] | RoB - RCT | Low | Low | Some | Low | Some | Some |
| Hamilton-Reeves et al., 2018 [49] | RoB - RCT | Low | Low | Some | Low | Some | Some |
| Kamo et al., 2020 [28] | RoB - RCT | Low | Some | Some | Low | Low | Some |
| Hardy et al., 2022 [35] | RoB - RCT | Low | Some | Some | Low | Low | Some |
| Nose et al., 2022 [53] | RoB - RCT | Low | Low | Low | Low | Some | Low |
| Tsutusi et al., 2024 [52] | RoB - RCT (Prospective-controlled, but randomised) | Some | Some | Low | Low | Some | Some |

Supplemental Table 2 - Risk of bias assessment for Randomised Controlled studies using the Cochrane-RoB Scale.

Supplemental Table 2 - Risk of bias assessment for randomised trials studies using the Revised Cochrane risk-of-bias tool (RoB 2).

| **Year** | **Number of Patients** | **Type of Surgery** | **Type of Study** | **Measures Used** | **Key Findings** | **Timings of Assessments** | **Included in Meta-Analysis** | **Reference** |
| --- | --- | --- | --- | --- | --- | --- | --- | --- |
| 1999 | 24 | Colonic resections for IBD | RCT – Assessing impact of perioperative growth hormone treatment on post-operative muscle function | Total muscle strength (TMS)  DEXA Imaging – Lean tissue mass (LTM) | - Significant reductions in mean TMS in both groups at 10d post-operatively relative to baseline. Significantly greater reduction in placebo group (17.1%) relative to intervention (7.6%). - At 30d post-operatively TMS still decrease relative to post-operative values. 13.1% in placebo group and 4.1% in intervention group. - At 7d post-operatively there was a reduction in LTM of 9.9% in placebo and 4.4% in intervention groups relative to pre-operative baseline. | 2d pre-operatively and 2d, 10d, 30d and 90d post-operatively | N | Kissmeyer-Nielsen et al., 1999 [59] |
| 2003 | 48 | Colorectal resections | RCT – Assessing impact of carbohydrate-rich drinks on post-operative muscle wasting | Muscle Strength – Quadriceps maximal isometric strength | - The use of carbohydrate-rich drinks reduced the degree of quadriceps strength loss post-operatively. | 1d pre-operatively and 1w and 1m post-operatively | N | Henriksen et al., 2003 [48] |
| 2012 | 13 | Colorectal resections | Prospective observational | DEXA Imaging – Muscle mass | - After surgery, patients with cancer displayed significant reductions in lean leg mass. | 1w pre-operatively and 6w post-operatively | Y | Williams et al., 2012 [60] |
| 2015 | 54 | Oesophagectomy | Prospective observational | Bioelectrical impedance analyser (BIA) – Lean tissue mass (LTM) | - LTM decreased from 48.2kg to 42.2kg post-operatively. | Pre-operatively (timings not specified) and 1m, 3m and 6m post-operatively | Y | Ni Bhuachalla et al., 2015 [57] |
| 2016 | 72 | Living-donor liver transplantation | Prospective observational | Bioelectrical impedance analyser (BIA) – Skeletal muscle mass (SMM)  Handgrip strength (HGS) | - SMM declined post-operatively and did not recover to pre-operative baseline until 12m. - HGS decreased sharply at 1m post-operatively but recovered to pre-operative baseline at the 6m mark. | Pre-operatively (on admission) and 1m, 2m, 3m, 6m and 12m post-operatively. | N | Kaido et al., 2016 [36] |
| 2016 | 21 | Living-donor liver transplantation | Retrospective | 6-minute walk distance (6MWD)  Handgrip strength (HGS)  Isometric knee extensor muscle strength (IKEMS) | - Physical function parameters had not been fully recovered 4 weeks after LDLT. - HGS decreased from 21.4 kgf pre-operatively to 17.3 kgf 4w post-operatively. | Median 6d pre-operatively and 4w post-operatively. | N | Mizuno et al., 2016 [61] |
| 2017 | 89 | Cystectomy | Retrospective analysis | CT Imaging - Changes in psoas muscle area, skeletal muscle index (SMI) at L3 | - Reduction in SMI relative to baseline at 1m, 3m, 6m, 12m and 24m. | Pre-operatively (timing not specified), 1m, 3m, 6m, 12m and 24m post-operatively. | Y | Miyake et al., 2017 [37] |
| 2017 | 254 | Liver resection | Prospective observational | CT Imaging – Changes in total psoas muscle area at L3. | - 2.2% median decrease in total psoas area 7 days post-operatively. | Within 30d pre-operatively and 1w post-operatively. | Y | Otsuji et al.,  2017 [62] |
| 2017 | 110 | Gastrectomy | Prospective observational | CT Imaging – Total abdominal muscle area (TAMA). Patients with muscle wasting ≥10% were defined to have clinically relevant muscle wasting. | - Median loss of skeletal muscle mass was 5.7% - Muscle wasting seen in 31% of patients. - Muscle wasting associated with decreased QoL and physical function (handgrip strength) and increased complication rate, hospital stay. - Diabetes and advanced age risk factor for post-operative muscle wasting. | Within 1m pre-operatively and 1 week post-operatively. | N | Huang et  al., 2017 [9] |
| 2017 | 191 | Oesophagectomy | Randomised controlled trial – Assessing effects of prolonged enteral nutrition (EN) with supplemental eicosapentaenoic acid (EPA) on postoperative clinical and nutritional outcomes | Bioelectrical impedance analyser (BIA) – Lean body mass (LBM) | - There was no difference between the intervention or control in any outcomes post-operatively. - Decrease in LBM of 6.3% in intervention and 8.3% in control groups at 1m post-operatively. | Pre-operatively (timing not specified) and 1m, 3m and 6m post-operatively | N | Healy et al., 2017 [43] |
| 2018 | 29 | Cystectomy | RCT – Assessing impact of specialised immunonutrition (SIM) on post-operative muscle wasting following cystectomy | DEXA Imaging – Relative skeletal muscle index (RMSI) | - Loss of skeletal muscle in the intervention was 7.04% and 16.9% in the control group at 14d post-operatively. | Pre-operatively (timing not specified) and 2d, 14d and 30d post-operatively | Y | Hamilton-Reeves et al., 2018 [49] |
| 2018 | 36 | ABO-incompatible living-donor liver transplantation | Retrospective analysis | CT Imaging – L3/L4 Psoas muscle index (PMI) | - Relative to the pre-operative baseline the PMI on 7d was 95.3% and on 3w post-operatively was 90.7%. | Within 1m pre-operatively and 1w, 3w, 6m and 1y post-operatively | N | Chae et al., 2018 [63] |
| 2018 | 473 | Living-donor liver transplantation | Retrospective analysis | CT Imaging – L3/4 Psoas muscle index (PMI) | - The median PMI change from pre-operative baseline to 7d post-operatively was a -4.8%. | Within 1m pre-operatively and 1w, 3w, 6m and 1y post-operatively | Y | Chae at al., 2018 [64] |
| 2019 | 192 | Cancer resections (upper GI, colorectal and genitourinary) | Prospective observational | Handgrip strength (HGS)  Maximum tongue pressure (MTP) | - MTP and HGS decrease more significantly in the upper GI groups compared to the genitourinary groups. - In upper GI and colorectal groups there was a decrease in both MTP and HGS at 4d post-operatively. | Pre-operatively (timings not specified) and 4d post-operatively | N | Taniguchi et al., 2019 [65] |
| 2019 | 7 | Major elective colorectal surgery (in patients aged 65 and older) | Prospective observational | Ultrasound Imaging – Bilateral anterior thigh thickness: subcutaneous tissue thickness (BATT-SCR)  Handgrip strength (HGS)  Gait speed (m/s) | - At 1d post-operatively there was a median decrease of 42.7% in BATT-SCR and a median decrease of 55.1% at 7d post-operatively. - There was no significant change in handgrip strength. - Gait speed declined from a median of 0.89 m/s pre-operatively to a median of 0.83 m/s at 1w post-operatively. | 1d pre-operatively and 1d and 1w post-operatively | Y | Welch et al., 2019 [39] |
| 2019 | 457 | Liver donors | Retrospective | CT Imaging – L3/4 Psoas muscle index (PMI) | - 4.6-5.0% decrease in PMI at 7d post-operatively, which improved by 3m. | Within 1m pre-operatively and 1w and 3m post-operatively | Y | Kim et al.,  2019 [47] |
| 2019 | 138 | Living-donor liver transplantation | Retrospective analysis | CT Imaging – Skeletal muscle index (SMI) at L3 | - In males there was a slight decrease in post-operative SMI at 1m post-operatively, compared to females who displayed a slight increase. - At 1y post-operatively, both males and females showed a small increase in SMI relative to pre-operative baseline. | Pre-operatively (timing not specified) and 1m and 1y post-operatively | Y | Pravisani et al., 2019 [66] |
| 2019 | 37 | Hepatectomy | Non-randomised controlled trial – Assessing impact of pre-operative late evening snack (LES) on post-operative nutritional status | Bioelectrical impedance analysis (BIA) – Skeletal muscle volume (SMV)  Arm muscle circumference (cm) | - At 4w post-operatively skeletal muscle volume, relative to baseline, was 93.9% in the control group and 97.5% in intervention group. - At 4w post-operatively arm circumference, relative to baseline, was 94.7% in the control group and 97.0% in intervention group. - Skeletal muscle volume was significantly lower in the control group compared to the intervention group at 4w and 12w after surgery. | 2w pre-operatively and 1w, 4w and 12w post-operatively | Y | Kobayashi et al., 2019 [67] |
| 2020 | 159 | Oesophagectomy | Retrospective | Bioelectrical impedance analysis (BIA) – Muscle mass (MM) | - Median rate of MM loss was 4.38% at hospital discharge. - Patients with increased MM loss had significantly poorer outcomes compared to those with a decrease MM loss. | Within 1w pre-operatively and at discharge (mean days 19.7/24.2 depending on group) | N | Shimoda et al., 2020 [44] |
| 2020 | 72 | Oesophagectomy | Retrospective | CT Imaging – L3 total psoas major muscle index (TPI) | - Median of 4.4% decrease in TPI at 3d post-operatively. - Psoas muscle area loss associated with worse survival rates. | Within 1w pre-operatively and 3d post-operatively | Y | Maeda et  al., 2020 [5] |
| 2020 | 89 | Pancreatic cancer resections | Retrospective | CT Imaging – Skeletal muscle mass (SMM) at L3 | - SMM decreased throughout the 12-week period after resection. - The restoration of muscle mass at 24 weeks after resection may be an independent prognostic factor for survival following pancreatic cancer resections | Pre-operatively (time not specified) and 4w, 12w and 24w post-operatively | Y | Lee et al., 2020 [29] |
| 2020 | 33 | Liver transplantation | Randomised controlled trial – Assessing effects of oral nutritional formula-enriched b-hydroxy-b-methyl-  butyrate (HMB) on post-operative sarcopenia. | Handgrip strength (HGS)  CT Imaging – L3 Skeletal muscle index (SMI) | - Those in HMB group had significantly higher values for HGS at 1m post-operatively compared to control group. - In the HMB group there was an increase in SMI at 1m and 2m post-operatively, compared to decreases in the control group. | Within 1m (CT) and 2w (HGS) pre-operatively and 1m and 2m post-operatively | Y | Kamo et al., 2020 [28] |
| 2021 | 128 | Liver resection | Retrospective | CT Imaging – L3 Psoas Muscle Index (PMI) | - Post-operative loss of PMI observed in 52% of patients. - Mean PMI showed a mean 1.6% decrease postoperatively compared to pre-operative baseline. | Within 6w pre-operatively and mean 7d post-operatively | Y | van Wijk et  al., 2021 [68] |
| 2021 | 28 | Pancreaticoduodenectomy | Retrospective | CT Imaging – L3 skeletal muscle mass (cm^2^) | - Muscle mass increased from 118.3 pre-operatively to 124.5 cm^2^ at 7d post-operatively. | Mean 16d pre-operatively and 7d post-operatively | N | Cha et al., 2021 [69] |
| 2021 | 128 | Liver resections | Propensity score matching analysis – Comparing change in post-operative psoas muscle index (PMI) of laparoscopic versus open liver resections | CT Imaging – L3 Psoas muscle index (PMI) | - PMI was significantly decreased post-operatively in the open liver resection group with a mean change of -6.61% relative to laparoscopic liver resection -3.37%. - Mean change in PMI post-operatively was -5.0% across the entire cohort. - 13.3% of patients had at least 11.5% loss of PMI | Within 1m pre-operatively and 1m post-operatively | Y | Gau et al., 2022 [70] |
| 2021 | 71 | Pancreaticoduodenectomy | Retrospective | CT Imaging – L3 skeletal muscle area (SMA) | - Median SMA decrease of 14% at 10d post-operatively. - Elevated change of urea to creatinine ratio (CUCR) reflects skeletal muscle wasting in postoperative surgical patients. | Within 1w pre-operatively and 7-10d post-operatively | Y | Duan et al., 2021 [71] |
| 2021 | 152 | Oesophagectomy | Retrospective analysis | CT Imaging – Total psoas area (TPA) at L3 | - TPA increased from 1454.96 mm^2^ pre-operatively to 1474.41 mm^2^ at 7d post-operatively. | Within 1m pre-operatively and 7d post-operatively | Y | Matsui et al., 2021 [7] |
| 2022 | 69 | Gastrointestinal (gastric, colorectal, liver and pancreatic) cancer resections | Retrospective observational | Bioelectrical impedance analysis (BIA) – Skeletal muscle index (SMI)  Handgrip strength (HGS) | - 7.5% decrease in mean SMI at 10d post-operatively. - 4.9% decrease in mean HGS at 10d post-operatively. | 1d pre-operatively and 10d post-operatively | Y | Tanaka et al., 2022 [42] |
| 2022 | 15 | Open colorectal resections | Prospective interventional trial – Assessing impact of neuromuscular electrical stimulation (NMES) on post-operative muscle mass and function | Ultrasound Imaging – Cross-sectional area (CSA) and muscle thickness (MT) of vastus lateralis | - 9.16% loss of CSA in control limb compared with 2.76% loss in the intervention limb - 10.35% reduction in knee extensor strength compared to 19.99% reduction in the intervention limb | 1d pre-operatively and 5d post-operatively | Y | Hardy et  al., 2022 [35] |
| 2022 | 20 | Oesophagectomy | Prospective observational | CT Imaging – L3 skeletal muscle index (SMI) | - There was a 3.73% decrease in SMI 1w post-operatively. | Within 30d pre-operatively and 1w and 6m post-operatively | Y | Matsui et al., 2022 [72] |
| 2022 | 73 | Oesophagectomy | Randomised controlled trial (RCT) – Assessing effects of ghrelin administration on post-operative muscle wasting | CT Imaging – Cross-sectional area (CSA) of T12 erector spinae muscle | - Ghrelin administration for 7d post-operatively significantly reduced rates of muscle loss compared to placebo. - At 7d post-operatively there was a CSA loss of 2.8% in the high-dose ghrelin group, 4.9% in the low-dose ghrelin group and 8.5% in the placebo group relative to pre-operative baseline. | Within 2w pre-operatively and 7d post-operatively | N | Nose et al., 2022 [53] |
| 2022 | 165 | Gastrectomy | Retrospective – Assessing effects of amino acid nutrition on postoperative skeletal muscle loss | Bioelectrical impedance analysis (BIA) – Skeletal muscle index (SMI) | - The percentage of skeletal muscle loss at 7 days post-operatively was less in the amino acid group (-4.1%) compared to the control group (-6.5%). - At 1 month post-operatively there was similar post-operative skeletal muscle loss between the intervention (-4.14%) and control groups (-4.56%). | Pre-operatively (timing not specified) and 1w and 1m post-operatively | N | Kikuchi et al., 2022 [73] |
| 2022 | 78 | Oesophagectomy | Prospective observational | Bioelectrical impedance analysis (BIA) – Skeletal muscle index (SMI) | - At 4w post-surgery there was a -3.42% change in SMI relative to pre-operative baseline. | 1d pre-operatively and 1w, 4w and 12w post-operatively | Y | Wang et al., 2022 [46] |
| 2023 | 63 | Pancreatic resections | Prospective observational | Ultrasound Imaging – Diameters of biceps brachii, rectus femoris and vastus intermedius. | - 39% of patients displayed SRML (≥10% decrease in one arm and leg muscle at 7d post-operatively). | 1d pre-operatively and 3d, 7d and 10d post-operatively. | Y | Hogenbirk  et al., 2023 [6] |
| 2023 | 135 | Oesophagectomy | Retrospective | CT Imaging – L3 psoas muscle index (PMI) | - 38% of patients had a decrease in PMI 1w post-operatively. | Pre-operatively (timing not specified) and 1w, 1y and 3y post-operatively | Y | Nishimura et al., 2023 [74] |
| 2023 | 202 | Gastrectomy | Retrospective | Bioelectrical impedance analysis (BIA) – Skeletal muscle mass | - Following gastrectomy there were decreases of 3.49% and 5.57% in skeletal muscle mass at 1w and 1m post-operatively respectively. | Within 1m pre-operatively and 1w and 1m post-operatively | N | Osaki et al., 2023 [75] |
| 2024 | 114 | Oesophagectomy | Retrospective | CT Imaging – L3 psoas muscle area (PMA) | - Psoas muscle area was significantly higher 1m and 1y post-operatively in patients receiving amino acid enriched nutritional therapy | Pre-operatively (timing not specified) and 1m and 1y post-operatively. | Y | Okamoto et al., 2024 [51] |
| 2024 | 39 | Liver transplant recipients | Prospective observational | Bioelectrical impedance analysis (BIA) – Skeletal muscle index (SMI) | - Sarcopenia significantly increased one month after liver transplant surgery. - There was a decrease in SMI of 16.7% at 1m and 12.8% at 3m post-operatively. | Pre-operatively (timing not specified) and 1m and 3m post-operatively. | Y | Khodashahi et al., 2024 [58] |
| 2024 | 30 | Living-donor liver transplantation | Prospective controlled trial – Assessing impact of electrical muscle stimulation (EMS) on perioperative muscle mass changes | CT Imaging – Psoas muscle index (PMI) and skeletal muscle index (SMI) at level of L3 and quadriceps muscle area | - The use of EMS prevented a postoperative reduction in the quadriceps muscles and the PMI. | Pre-operatively (timing not specified) and 1m post-operatively | N | Tsutsui et al., 2024 [52] |
| 2024 | 36 | Major elective gastrointestinal surgeries | Prospective non-randomised interventional cohort study – Assessing impact of pre-operative multimodal prehabilitation programme on post-operative sarcopenia | Handgrip strength (HGS)  Mid-arm muscle circumference | - No significant changes in HGS or mid-arm muscle circumference were noted during the perioperative period. | Within 1m pre-operatively and 2w and 4w post-operatively. | N | Koh et al., 2024 [76] |
| 2025 | 441 | Gastrectomy | Retrospective analysis | CT Imaging – L3 Skeletal muscle index (SMI) | - At 1m post-operatively there was a 5.7% decrease in SMI relative to pre-operative baseline. | Pre-operatively (timing not specified) and 1m post-operatively | Y | Hashimoto et al., 2025 [77] |
| 2025 | 113 | Gastrectomy | Retrospective analysis | Bioelectrical impedance analysis (BIA) – Skeletal muscle index (SMI) | - At 1m post-operatively those taking oral nutritional supplementation had a post-operative muscle loss of -3.6% and those on standard therapy had a muscle loss of -4.8%. | Pre-operatively (timing not specified) and 1m post-operatively | Y | Kikuchi et al., 2025 [50] |
| 2025 | 52 | Major abdominal cancer surgery (hepato-pancreato-biliary or gynaecological) | Prospective observational | Ultrasound Imaging – Rectus femoris cross-sectional area (RFCSA)  Handgrip strength (HGS)  Sniff nasal inspiratory pressure (SNIP)  Actigraphy monitoring (mean activity count) | - A significant reduction in RFCSA was observed in 50% of patients, with a median decrease of 10.2% at 1w post-operatively. - At 6w post-operatively there was a mean 12% decrease in RFCSA. - Mean HGS showed a significant decline of -10.7% at 1w post-operatively. - Mean SNIP also showed a significant decline of -21.7% at 1w post-operatively. - Patients with <10% RFCSA reduction demonstrated significantly higher activity than those with a >10% reduction. - Postoperative activity levels correlated with RFCSA preservation. | 1d pre-operatively and 1w and 6w post-operatively | Y | Dudas et al., 2025 [34] |
| 2025 | 198 | Gastrectomy | Retrospective analysis | CT Imaging – L3 Skeletal muscle index (SMI) | - The mean change in SMI at 1m post-operatively was -5.95%. - Skeletal muscle loss was associated with a higher post-operative complication rate. | 1w pre-operatively and 1m post-operatively | Y | Li et al., 2025 [45] |
| 2025 | 335 | Gastrectomy | Prospective observational | CT Imaging – Skeletal muscle index (SMI) and skeletal muscle density (SMD) at L3 | - Excessive SMI and SMD loss were associated with adverse clinical outcomes. - Median decrease of 5.7% of SMI and decrease of 14.4% for SMD at 7d post-operatively relative to baseline. | Within 30d pre-operatively and 7d post-operatively. | Y | Zhao et al., 2025 [78] |

Supplemental Table 3 – Characteristics of included studies, listed in chronological order of publication.
